# Aberrant P-cadherin expression is associated to aggressive feline mammary carcinomas

**DOI:** 10.1186/s12917-014-0270-z

**Published:** 2014-11-26

**Authors:** Ana Catarina Figueira, Catarina Gomes, Joana Tavares de Oliveira, Hugo Vilhena, Júlio Carvalheira, Augusto JF de Matos, Patrícia Dias Pereira, Fátima Gärtner

**Affiliations:** Escola Universitária Vasco da Gama (EUVG), Av. José R. Sousa Fernandes, Campus Universitário de Lordemão, Bloco B, Lordemão, 3020-210 Coimbra, Portugal; Instituto de Ciências Biomédicas Abel Salazar, Universidade do Porto (ICBAS-UP), Rua de Jorge Viterbo Ferreira No. 228, 4050-313 Porto, Portugal; Instituto de Patologia e Imunologia Molecular da Universidade do Porto (IPATIMUP), Rua Dr Roberto Frias s/n, 4200-465 Porto, Portugal; Hospital Veterinário do Baixo Vouga (HVBV), Estrada Nacional 1, 355, Segadães, 3750-742 Águeda, Portugal; Centro de Ciência Animal e Veterinária (CECAV), Universidade de Trás-os-Montes e Alto Douro (UTAD), Quinta de Prados, 5000-801 Vila Real, Portugal; Centro de Investigação em Biodiversidade e Recursos Genéticos (CIBIO), Universidade do Porto (UP), Rua Padre Armando Quintas, 4485-661 Vairão, Portugal; Centro de Estudos de Ciência Animal (CECA), Instituto de Ciências e Tecnologias Agrárias e Agro Alimentares (ICETA), Universidade do Porto (UP), Rua D. Manuel II, ap° 55142, 4051-401 Porto, Portugal

**Keywords:** Feline mammary tumours, Invasion, P-cadherin, E-cadherin

## Abstract

**Background:**

Cadherins are calcium-dependent cell-to-cell adhesion glycoproteins playing a critical role in the formation and maintenance of normal tissue architecture. In normal mammary gland, E-cadherin is expressed by luminal epithelial cells, while P-cadherin is restricted to myoepithelial cells. Changes in the expression of classical E- and P-cadherins have been observed in mammary lesions and related to mammary carcinogenesis. P-cadherin and E-cadherin expressions were studied in a series of feline normal mammary glands, hyperplastic/dysplastic lesions, benign and malignant tumours by immunohistochemistry and double-label immunofluorescence.

**Results:**

In normal tissue and in the majority of hyperplastic/dysplastic lesions and benign tumours, P-cadherin was restricted to myoepithelial cells, while 80% of the malignant tumours expressed P-cadherin in luminal epithelial cells. P-cadherin expression was significantly related to high histological grade of carcinomas (*p* <0.0001), tumour necrosis (*p* = 0.001), infiltrative growth (*p* = 0.0051), and presence of neoplastic emboli (*p* = 0.0401). Moreover, P-cadherin positive carcinomas had an eightfold likelihood of developing neoplastic emboli than negative tumours. Cadherins expression profile in high grade and in infiltrative tumours was similar, the majority expressing P-cadherin, regardless of E-cadherin expression status. The two cadherins were found to be co-expressed in carcinomas with aberrant P-cadherin expression and preserved E-cadherin.

**Conclusions:**

The results demonstrate a relationship between P-cadherin expression and aggressive biological behaviour of feline mammary carcinomas, suggesting that P-cadherin may be considered an indicator of poor prognosis in this animal species. Moreover, it indicates that, in queens, the aberrant expression of P-cadherin is a better marker of mammary carcinomas aggressive behaviour than the reduction of E-cadherin expression. Further investigation with follow-up studies in feline species should be conducted in order to evaluate the prognostic value of P-cadherin expression in E-cadherin positive carcinomas.

## Background

Cadherins are calcium-dependent cell-to-cell adhesion glycoproteins with critical roles in the formation and maintenance of normal tissue architecture [1-4], including in normal mammary gland [4-7]. E-cadherin (epithelial) and P-cadherin (placental), are the best characterised members of the cadherin superfamily [8], similarly expressed in normal mammary epithelium of different species, namely human [4,6,9-13], canine [14-17] and feline [18-20]: E-cadherin is expressed by luminal epithelial cells while P-cadherin is restricted to myoepithelial cells. Changes in their expression have been observed in mammary tumours and related to mammary carcinogenesis, both in humans [5,9,11-13,21-27], dogs [14,16,17,28-30], as well as in cats [18-20,31,32]. Loss of expression and/or abnormal function of E-cadherin increase the ability of cells to invade neighbouring tissues, thus favouring mammary tumour progression and spread [4,13,33-35], whereas overexpression of P-cadherin is related to increased cell proliferation, motility, invasiveness, and metastatic progression, thus being considered an invasion-promoting protein in breast cancer [5,22,25,26,36-38]. Moreover, P-cadherin inhibition has anti-tumoural and anti-metastatic effects, suggesting that interrupting the P-cadherin signalling pathway may be a novel therapeutic approach for breast cancer [5,39,40].

Mammary gland tumours are the third most common neoplasm in queens and have been proposed as excellent model for the study of human mammary carcinogenesis, due to histopathological and clinical similarities with human breast cancer [41,42]. As opposed to the large body of knowledge on E- and P-cadherin expressions in human breast carcinomas, there are only a few studies of these molecules in feline mammary tumours [18-20,31,32,43], and their role is still poorly understood in this species. Studies on E-cadherin protein in feline mammary tumours demonstrated its reduction or absent expression in carcinomas when compared to benign lesions [18,19,31,32]. The prognostic value of this molecule in feline carcinomas is still controversial: while no statistically significant association between E-cadherin expression and tumour histological grade has been found [31], one study demonstrated a negative correlation between E-cadherin expression and regional lymph node metastases at the time of diagnosis [31], however another revealed no correlation between the expression of E-cadherin and survival, recurrence or metastases [19].

To the best of our knowledge, only one study addressed the P-cadherin expression in feline mammary tumours, demonstrating that the protein is aberrantly expressed by neoplastic epithelial cells in malignant tumours and significantly associated to high grade carcinomas [20].

The purposes of this study were to examine the expression of P-cadherin in a series of normal mammary gland tissues and spontaneous hyperplastic and tumour mammary lesions, and to determine its relationship with the expression of E-cadherin as well as tumour clinicopathological features with recognized prognostic value [44-49], namely neoplastic intravascular emboli and lymph node metastases.

## Methods

### Tissue samples

Samples from 75 queens with naturally occurring mammary lesions, surgically excised with curative intents, and nine normal mammary glands (obtained from queens that were humanely euthanized for reasons not related to a neoplastic disease) were included in this study. In each case an informed consent was granted by the owners. All specimens were fixed in 10% neutral buffered formalin. After tissue dehydration and embedding in paraffin wax, sequential 2 μm sections were cut from each block. One section was stained with haematoxylin and eosin (H&E) for routine histological examination and diagnosis, and subsequent sections were used for immunohistochemical studies. When available, local and regional lymph nodes were processed and examined for the presence of metastases.

The histological classification of tumours was independently performed by three observers (ACF, PDP and FG) based on the criteria of the World Health Organization (WHO) for the histological classification of mammary tumours of domestic animals [50].

Carcinomas were graded in accordance with the Nottingham grading system for human breast carcinomas [51]. Grading was based on the assessment of three morphological features: degree of tubule formation, nuclear pleomorphism, and mitotic counts, and tumours were classified as grade I (well differentiated), grade II (moderately differentiated) and grade III (poorly differentiated) [51]. Variables with known prognostic value, such as the mode of growth (infiltrative or expansive), tumour largest diameter (<2 cm, 2–3 cm, >3 cm), presence of necrosis, skin ulceration, lymph node metastases, and presence of intravascular neoplastic emboli [41,44], were also recorded.

### Evaluation of P-cadherin and E-cadherin immunohistochemistry labelling

Immunohistochemistry (IHC) was performed using a polymer based system (Novolink Max Polymer Detection System, Product No: RE7280-K Leica Biosystems, Newcastke, UK), according to the manufacturer’s instructions. Sections were dewaxed in xylene, rehydrated through graded alcohols and treated with extran for 10 minutes in microwave oven for antigen retrieval. Endogenous peroxidase activity was blocked by treating the sections with hydrogen peroxide 3% in methanol for 10 minutes and rinsed in Tris-buffered saline (TBS, pH 7.6, 0.5 M). Sections were incubated overnight at 4°C in a humid chamber with a specific mouse anti-human monoclonal antibody against P-cadherin (clone 56, BD Transduction Laboratories, Lexington, Kentucky, USA) directed at the extracellular domain of this adhesion molecule, and a specific mouse anti-monoclonal antibody against E-cadherin (clone 4A2C7, Zymed/Invitrogen, Camarillo, CA, USA) that recognizes the cytoplasmic domain of this molecule. The antibodies were diluted 1:50 in TBS with 5% bovine serum albumin (BSA). Labelling was visualized with 3,3’-diaminobenzidine (DAB) incubated at room temperature and sections were then counterstained with Mayer’s haematoxilin, dehydrated and mounted. For negative controls, the primary antibody was replaced by TBS. Sections of feline normal mammary gland were used as positive controls. In the sections of mammary lesions, adjacent normal mammary tissues or skin were also used as internal positive controls.

In the lymph nodes sections, additional immunohistochemical analysis was performed in order to determine the presence of tumour cell micrometastases, as suggested by Matos *et al*. [52]. However, we used the antibody anti-pancytokeratin AE1/AE3 and anti-p63 protein, since p63 protein is a sensitive and highly specific marker of myoepithelial cells [53].

Assessment of P-cadherin expression was based on a semi quantitative analysis, according to the percentage of immunoreactive luminal epithelial cells with membranous and/or cytoplasmic patterns, and graded as 0 - <10%, 1 - 10-25%, 2 - 26-50% and 3 - >50%. For statistical analysis, cases with less than 10% positive luminal epithelial cells were considered negative and those with ≥10% stained cells were considered positive [16,20].

E-cadherin expression was assessed semi quantitatively in accordance with the percentage of immunoreactive luminal epithelial cells with membranous labelling, and graded as 0 - <25%, 1 - 25-50%, 2 - 51-75% and 3 - >75% [54]. Cases with >75% stained cells were considered to have preserved expression and those with ≤75% positive cells as having reduced expression of E-cadherin [55].

To evaluate the combined expression of P-cadherin and E-cadherin, samples were grouped according to the expression of both molecules as P^+^E^+^, P^+^E^−^, P^−^E^+^, and P^−^E^−^, (P^+^ = P-cadherin positive; P^−^ = P-cadherin negative; E^+^ = Preserved E-cadherin; E^−^ = Reduced E-cadherin) [56].

### Evaluation of P-cadherin and E-cadherin double-labelling immunofluorescence

Double-label immunofluorescence (DIF) was performed for simultaneous visualization of P- and E-cadherin expressions. Tissue sections of normal mammary gland, hyperplastic/dysplastic lesions, benign and malignant mammary tumours were selected and sections with neoplastic intravascular emboli and lymph node metastases were also included.

Tissue sections were dewaxed in xylene, rehydrated through a series of graded alcohols and treated with extran for 10 minutes in microwave oven for antigen retrieval. Then, tissues sections were blocked with 10% BSA for 20 minutes, followed by incubation with the primary antibodies mouse anti P-cadherin (mouse anti-human monoclonal antibody against P-cadherin, clone 56, BD Transduction Laboratories, Lexington, Kentucky, USA) and rabbit anti E-cadherin (rabbit anti-human monoclonal antibody against E-cadherin, clone 24E10, Cell Signaling Technology, MA, USA) diluted 1:50 and 1:100, respectively, in 5% BSA for two hours in a wet chamber. After washing in phosphate buffered saline (PBS), slides were incubated with Alexa Fluor® 488 goat anti-mouse IgG (A11029, Life Technology, Carlsbad, CA, USA) and Alexa Fluor® 594 goat anti-rabbit IgG (A11037, Life Technology, Carlsbad, CA, USA) secondary antibodies diluted 1:500 in 5% BSA, for one hour. Washes were performed with PBS and slides incubated with 4,6-diamidine-2-phenylindolendihydrochoride (DAPI) 100 μg/mL for 15 minutes. Slides were mounted in glycerol-based Vectashield medium (Vector, Burlingame, CA, USA).

Immunostained sections were analyzed by fluorescence microscopy (Zeiss Imager Z1 microscope) with appropriated filters. Separate images for Alexa 488 and Alexa 594 were captured at ×200 magnification and then merged to allow for the visualization of P-cadherin and E-cadherin double immunostaining. For negative controls, the primary antibody was replaced by PBS.

### Statistical methods

Data was organized in contingency tables and the likelihood ratio chi-square test of associations was used to determine the significance of the relationship between the expression of the cadherins and the tumours’ clinicopathological parameters. Whenever biologically consistent, 2×2 tables of contingency were built and Fisher’s exact test was performed. The odds ratio was calculated to estimate the relative risk of lymph node metastasis and neoplastic intravascular emboli in tumours expressing P- and E-cadherin molecules, with a confidence interval of 95%. All statistical analysis was performed using SAS/STAT, 1989 (SAS Institute Inc., Cary, NC, USA) [57] and, in all instances, *p <*0*.*05 was considered to be statistically significant.

## Results

The present study comprised 9 normal mammary gland samples, 13 hyperplastic/dysplastic lesions (7 fibrocystic diseases and 6 fibroadenomatous changes), 10 benign tumours (7 simple adenomas and 3 fibroadenomas) and 60 malignant tumours (32 tubulopapillary carcinomas, 16 solid carcinomas, 4 cribriform carcinomas, 6 mucinous carcinomas and 2 carcinosarcomas). Seven malignant tumours were grade I, 25 grade II and 28 grade III. Twenty-one carcinomas (36.21%) had neoplastic intravascular emboli and, within the 35 cases where lymph nodes were available, 18 (51.43%) had metastases.

### P-Cadherin and E-cadherin expression by immunohistochemistry

**Figure 1:**
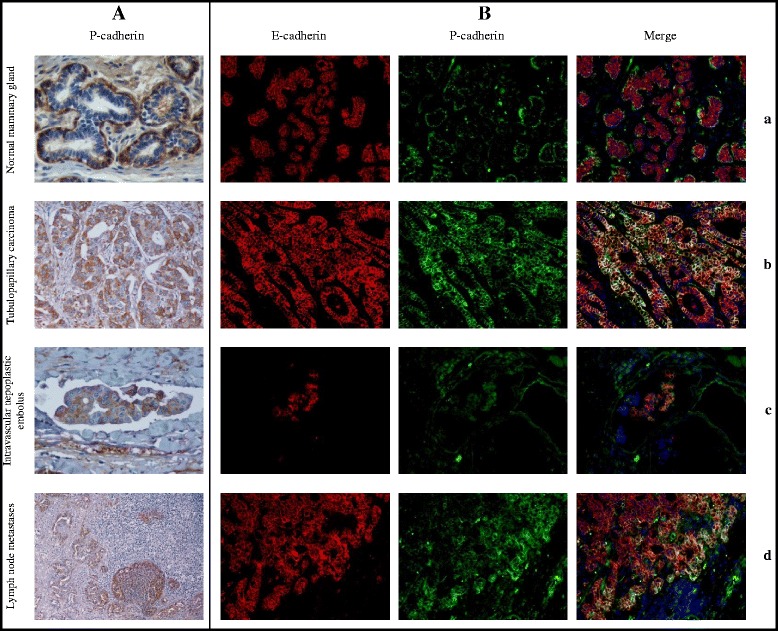
**P- and E-cadherin expression by immunohistochemistry (IHQ) and by double-label immunofluorescence (DIF) in feline mammary tissue. A**. P-cadherin immunohistochemistry (IHQ) - (a) Normal mammary gland with strong expression of P-cadherin by myoepithelial cells and lack of immunoreactivity in luminal epithelial cells. IHC. x400; (b) Strong P-cadherin expression in high atypical neoplastic cells from a grade III tubulopapillary carcinoma. IHC. x200; (c) Neoplastic intravascular embolus showing strong P-cadherin expression. IHC. x400; (d) Lymph node metastases showing P-cadherin expression by neoplastic cells. IHC. x100. **B**. P- and E-cadherin double-label immunofluorescence (DIF) - (a) Normal mammary gland tissue with P-cadherin in myoepithelial cells (green colour) and E-cadherin in luminal epithelial cells (red colour). DIF. x200; (b) Tubulopapillary carcinoma with tumour cell populations co-expressing P-cadherin and E-cadherin (yellow colour). DIF. x200; (c) Neoplastic intravascular embolus. DIF. x200. and (d) lymph node metastatic cells. DIF. x200. with a pattern of expression similar to primary malignant neoplasia, with tumour cell populations co-expressing P-cadherin and E-cadherin (yellow colour).

In normal mammary tissue, the expression of P-cadherin was restricted to myoepithelial cells surrounding lobular and ductal structures (Figure 1Aa). However, in lobules with secretory activity P-cadherin was present in the cytoplasm of luminal epithelial cells as well as in the secretion. A similar staining pattern to normal mammary tissue was evident in the majority (84.62%) of hyperplastic mammary lesions, although two fibroadenomatous changes exhibited P-cadherin expression in 10 to 25% of luminal epithelial cells. In benign tumours, P-cadherin was restricted to myoepithelial cells in most cases (70%). All 7 simple adenomas were P-cadherin negative while the 3 fibroadenomas were positive, one with 10-25% and the other two with 26-50% positive cells.

Within the 60 malignant tumours, 48 (80%) had aberrant luminal epithelial P-cadherin expression (Figure 1Ab), most noticeable in the tumour periphery, in invasive clusters, in tubulopapillary areas and in the most atypical cells (large cells, with bizarre shapes, large nucleus, multiple nucleolus). In mucinous carcinomas, the cells surrounding mucus were P-cadherin positive although the mucus itself was negative. P-cadherin expression was also observed in squamous cells, particularly in the most undifferentiated basal cells, and in the mesenchymal cells component of carcinosarcomas.

P-cadherin expression was analysed in the neoplastic intravascular emboli of 16 malignant tumours (in 5 cases no representative sections for immunohistochemical evaluation were available) and 18 lymph node metastases. Amongst the former, 13 (81.25%) were P-cadherin positive (Figure 1Ac) and 17 (94.4%) of the later were also P-cadherin positive (Figure 1Ad).

E-cadherin was expressed at the membrane of more than 75% acinar and ductal luminal epithelial cells of normal mammary gland samples, while myoepithelial cells were E-cadherin negative. Eleven (84.6%) hyperplastic lesions preserved this pattern of expression, while 2 cases of fibrocystic disease had less than 75% positive cells. Benign tumours preserved E-cadherin expression by luminal epithelial cells, except in one simple adenoma where only 51-75% of the epithelial cells were stained.

E-cadherin immunohistochemical expression was reduced in almost half (46.67%) of the malignant tumours. Five of the 6 mucinous carcinomas showed reduced E-cadherin expression, most obvious in the areas with mucus secretion; the mucus itself did not stain for E-cadherin. Squamous cells stained positive while mesenchymal and myoepithelial cells were negative to this protein.

Intravascular emboli of 17 malignant tumours were evaluated for E-cadherin expression (in 4 cases it was not possible to obtain representative sections for evaluation) and the protein was expressed by more than 75% cells in 13 cases (76.5%). More than half (55.6%) of the 18 lymph node metastases had more than 75% cells expressing E-cadherin.

### P-cadherin and E-cadherin expression by double-labelling immunofluorescence

The P-cadherin and E-cadherin double-labelling immunofluorescence analysis demonstrated that they were expressed in normal, hyperplastic and benign mammary tissues in different cell types, with luminal epithelial cells expressing E-cadherin, while myoepithelial/basal cells expressed P-cadherin (Figure 1Ba). In carcinomas it was possible to observe aberrant P-cadherin expression by luminal epithelial cells, frequently co-expressed with E-cadherin (Figure 1Bb), particularly at the peripheral invasive front. The same expression pattern was observed in the intravascular neoplastic emboli (Figure 1Bc) and in lymph node metastases (Figure 1Bd).

### Relationship between the expression of P-cadherin, E-cadherin and clinicopathological parameters

Benign and malignant tumours diverged significantly with respect to both P- and E-cadherin immunoexpression, with most of the malignant tumours (80%) overexpressing P-cadherin and the vast majority of the benign tumours (90%) preserving the expression of E-cadherin (Table 1). When the combined expression of P- and E-cadherin was considered, most (60%) of the benign tumours were P^−^/E^+^ while the most common pattern in malignant tumours was P^+^/E^−^ (41.67%).

**Table 1:** **Cadherin expression profiles of benign and malignant tumours**

**Tumour type**	**n**	**P-cadherin**		**E-cadherin**		**P-cadherin/E-cadherin**			
		**Negative**	**Positive**	**Preserved**	**Reduced**	**+/+**	**+/−**	**−/+**	**−/−**
Benign	10	7	3	9	1	3	0	6	1
Malignant	60	12	48	32	28	23	25	9	3
*p*		0.0029		0.0384		0.0027			

In malignant tumours, there was a statistically significant association between P-cadherin overexpression and higher histological grade, presence of necrosis, infiltrative mode of growth, and neoplastic intravascular emboli (Table 2). In the majority of malignant tumours, the expression of P-cadherin by neoplastic intravascular emboli and lymph node metastases was similar to that observed in corresponding primary tumours (81.25%, *p* = 0.0012 and 77.78%, *p* = 0.0008, respectively). Furthermore, the expression of P-cadherin was associated to a nearly 8.5 odds ratio for vascular invasion.

**Table 2:** **Cadherin expression profiles and clinicopathological parameters of malignant tumours**

**Clinicopathological parameters**	**n**	**P-cadherin**		**E-cadherin**		**P-cadherin/E-cadherin**			
		**Negative**	**Positive**	**Preserved**	**Reduced**	**+/+**	**+/−**	**−/+**	**−/−**
Histological type									
Tubulopapillary carcinoma	32	8	24	16	16	11	13	5	3
Solid carcinoma	16	3	13	9	7	6	7	3	0
Cribriforme carcinoma	4	1	3	4	0	3	0	1	0
Mucinous carcinoma	6	0	6	1	5	1	5	0	0
Carcinosarcoma	2	0	2	2	0	2	0	0	0
*p*		NS		0.0243		NS			
Histological grade*									
Grade I	7	7	0	5	2	0	0	5	2
Grade II	25	2	23	12	13	10	13	2	0
Grade III	28	3	25	12	13	13	12	2	1
*p*		<0.0001		NS		<0.0001			
Mode of growth**									
Expansive	3	3	0	3	0	0	0	3	0
Infiltrative	56	6	48	28	28	23	25	5	3
*p*		0.0051		NS		0.0044			
Tumour largest diameter									
<2 cm	33	7	26	19	14	13	13	6	1
2–3 cm	10	2	8	7	3	5	3	2	0
>3 cm	17	3	14	6	11	5	9	1	2
*p*		NS		NS		NS			
Ulceration									
Absent	48	10	38	27	21	19	19	8	2
Present	12	2	10	5	7	4	6	1	1
*p*		NS		NS		NS			
Necrosis									
Absent	12	7	5	9	3	4	1	5	2
Present	48	5	43	23	25	19	24	4	1
*p*		0.0010		NS		0.0026			
Neoplastic intravascular emboli									
Absent	37	11	26	19	18	11	15	8	3
Present	21	1	20	12	9	11	9	1	0
*p*		0.0401		NS		NS			
Odds ratio		8.4615 (1.0071-71.0959)							
Lymph node metastases									
Absent	17	3	14	10	7	7	7	3	0
Present	18	0	18	10	8	10	8	0	0
*p*		NS		NS		NS			

NS -not significant.

*According to Elston & Ellis 1998 [51].

**In one case the margins were not included in sample.

The reduced expression of E-cadherin, although significantly related to the malignant histological tumour type (*p* = 0.0243), was not related with the other parameters; neither there was a statistical significant association between the expression of E-cadherin in primary tumours and in their neoplastic intravascular emboli and lymph node metastases.

The P-cadherin/E-cadherin combined expression patterns were significantly associated with histological grade of carcinomas, mode of growth and presence of necrosis. In fact, 71.4% of grade I tumours were P^−^/E^+^, while nearly 90% of grade II and III tumours (n = 53) were P^+^/E^−^ (n = 25, 47.2%) or P^+^/E^+^ (n = 23, 43.4%). All tumours with expansive growth were P^−^/E^+^, a staining pattern that was present in less than 10% of the infiltrative ones. Half of the tumours with necrosis were P^+^/E^−^, while the most common pattern of non-necrotic cases was P^−^/E^+^ (41.67%).

## Discussion

In the present work, feline normal mammary tissue presented E-cadherin expression in luminal epithelial cells and P-cadherin in myoepithelial cells of lobular and ductal structures, without any evidence of overlapping between the two cadherins, similarly to what is described in normal mammary gland tissue in humans [4,6,9-13], dogs [14-17] and cats [18-20]. In lactating or pseudo-lactating mammary tissue, P-cadherin immunoreactivity was observed in the cytoplasm of luminal epithelial cells and in luminal secretion, in line with data from previous studies in human breast [58] and canine mammary gland [59]. It is believed that P-cadherin is not just an adhesion molecule, also acting as a signalling protein involved in breast tissue remodelling, and that its soluble fragment present in human milk may result from the proteolysis of the extracellular domain [58,59].

In this series, a significant overexpression of P-cadherin by luminal epithelial cells was observed in carcinomas, when compared to benign mammary tumours. Furthermore, P-cadherin expression was positive in all mucinous carcinomas and carcinosarcomas a fact that points to a basal/myoepithelial cell histogenesis origin or line of differentiation of these tumour types [16,60,61], since P-cadherin is a well-recognized biomarker of basal mammary carcinomas in humans [6,62-65]. However, the association between P-cadherin expression and the histological type of carcinomas was not achieved in the present study, has not been yet demonstrated in humans [5] and is not consensual in other animal models such as dogs [16,56]. A significant association was found between P-cadherin expression and histological grade of carcinomas, as previously reported by other authors in human breast cancer [11,21,23,24,26,27,62,66-68] and canine mammary tumours [16,56], suggesting that this molecule may be regarded as a prognostic indicator of aggressiveness in feline mammary carcinomas. Moreover, P-cadherin expression was more evident in the most periphery cells of the tumour and in the invasive clusters, denoting its importance in invasion and re-enforcing its value as a marker of adverse behaviour in feline mammary carcinomas.

To the best of our knowledge, this is the first study addressing the association between the expression of P-cadherin and the invasive/metastatic capacity of feline mammary tumours. All tumours with lymph node metastases and all, except one, with evident neoplastic intravascular emboli were P-cadherin positive, which indicates that the aberrant expression of P-cadherin may constitute an important step in the invasion/metastatic process. This hypothesis is reinforced by the fact that P-cadherin positive tumours were 8.46 times more likely to invade vessels than negative tumours. However, this result must be regarded with caution, since the wide confidence interval (CI) reveals a low precision of the estimate. Although in human breast cancer P-cadherin overexpression has been associated with decreased survival and relapse-free intervals [36,64], other studies failed to find a significant association between P-cadherin and lymph node metastases at the time of diagnosis [11,21,27]; however, Gamallo *et al*. [26] found an association between P-cadherin expression and lymph node-positive breast tumours. In this study we failed to establish a relationship between P-cadherin expression and lymph node metastases. Our findings suggest that, in spite of the importance of this cadherin in infiltrative/invasive process (increased cell motility, vascular invasion), the arrest and establishment of neoplastic cells in sites of metastases may require the acquisition of other morphological and functional features, not so closely related to P-cadherin. To the best of our knowledge there are no survival studies in feline mammary tumours related with P-cadherin expression.

Nearly half of the carcinomas included in this series exhibited reduced E-cadherin expression, a significant difference compared to benign tumours, where only one case showed E-cadherin down-expression. An association was also established between specific histological types of carcinomas and the E-cadherin expression pattern. All cribriform carcinomas and carcinosarcomas showed preserved E-cadherin expression, while the majority of mucinous carcinomas had a reduced expression of the protein. In cats, E-cadherin expression has been documented to be reduced in tubulopapillary [19], cribriform and solid carcinomas [18], although the statistical significance of such associations has never been assessed. Moreover, several studies demonstrated that a reduced/loss membrane expression of E-cadherin is significantly associated with histological types of mammary tumours, namely lobular carcinomas in women [9,10,13,69] and solid carcinomas in bitches [15,17,54,56,70]. In our study, E-cadherin expression was not related to histological grade of carcinomas. In fact, the association between this two factors is not consensual both in humans [9,10,71] and dogs [54,56] and the studies performed in cats have not proved this association, to date [19,31].

In the present study, the expression of E-cadherin was not associated to the presence of neoplastic intravascular emboli and lymph node metastases, corroborating the human breast cancer [4,9,10,72-74] and feline mammary tumours [19,31] literature in which there is no consensus regarding the prognostic value of E-cadherin. Furthermore, and in accordance with previous studies [72,75], the pattern of E-cadherin expression differ between primary tumours and their lymph node metastases, reinforcing the concept that, during breast carcinoma progression, there is a dynamic and reversible modulation of the E-cadherin complex [4,76,77].

When analysing the combined expression of P- and E-cadherins, two different patterns emerged: while 90% of the benign tumours preserved the expression of E-cadherin, irrespective of the P-cadherin status, 80% of the malignant tumours exhibited an overexpression of P-cadherin, independently of the E-cadherin staining pattern. These results suggest that the abnormal expression of P-cadherin is a better indicator of the malignant potential of feline mammary neoplasm than the loss of E-cadherin. Furthermore, the combined expression of P- and E-cadherin was significantly associated with the histological grade of the tumours, with the majority (90%) of grade II and III carcinomas exhibiting a P^+^/E^+^ or P^+^/E^−^ immunophenotype. The fact that half of the grade III P^+^ malignant tumours were also E^+^ deserves the evaluation of the prognostic value of aberrant P-cadherin expression in a context of preserved E-cadherin expression through follow-up studies. Ribeiro *et al*. [74] hypothesized that, in breast cancer cells, the E- and P-cadherin co-expression could be involved in a more aggressive biological behaviour, and that the establishment of strong adhesion complexes is compromised by the interaction of both molecules at the cell membrane. Moreover, they demonstrate that E^+^/P^+^ cells have deregulated cadherin/catenin complexes at the cellular membrane, when compared with cells expressing only one of the cadherins and have a higher invasive capacity. In fact, P-cadherin overexpression in an E-cadherin wild type context leads to disruption of the interaction between E-cadherin and intracellular catenins, an alternative mechanism for cancer invasion [5,74,78,79]. The co-expression of both molecules was significantly correlated with high-grade, biologically aggressive, breast carcinomas and poor patient survival [5,39,74].

The association between P- and E-cadherin expression and four clinicopathological parameters with known prognostic value in feline mammary tumours, namely ulceration [46,47], necrosis [47], infiltrative growth [44], and tumour largest diameter [47-49], was also addressed in this study. Interestingly, the E- and P-cadherin immunostaining pattern observed in infiltrative carcinomas was very similar to the findings in grade III tumours, i.e. the majority expressing P-cadherin, and surprisingly, nearly half of the P^+^ infiltrative carcinomas preserved E-cadherin expression. This is in accordance with data from immunofluorescence analysis that revealed P- and E-cadherin co-expression, particularly at the malignant tumour periphery. When considered together (neoplastic intravascular emboli and/or lymph node metastases), none of the invasive tumours was P^−^/E^−^, suggesting that the simple reduction of E-cadherin, when not accompanied by an aberrant expression of P-cadherin, is insufficient for an invasive tumour behaviour. Furthermore, more than half of the P-cadherin positive tumours associated with vascular emboli and lymph node metastases were also E-cadherin positive. In fact, in P^+^/E^+^ malignant tumours, double immunofluorescence evidenced a co-expression of the two cadherins, a pattern also observed in the intravascular neoplastic emboli and in lymph nodes metastatic cells. These results reinforce the need for the study of the prognostic value of P-cadherin positivity in tumours that preserve E-cadherin expression in feline mammary species.

Besides its prognostic value, P-cadherin has been recently considered as a therapeutic target. A highly selective human monoclonal antibody against P-cadherin (PF-03732010, Pfizer, Inc) may constitute a novel anticancer therapy in high P-cadherin expressing tumours [40]. Furthermore, azurin is pointed as a therapeutic tool for poor-prognosis breast carcinomas overexpressing P-cadherin in a wild type E-cadherin context [39]. Within this scenario, the highly aggressive P-cadherin positive feline mammary tumours with preserved E-cadherin expression may benefit from one of these novel therapeutic approaches.

## Conclusions

The present study demonstrated an association between the aberrant expression of P-cadherin and a malignant phenotype, higher histological grade and invasive behaviour, suggesting that this protein may constitute a reliable independent biomarker of poor prognosis in feline mammary tumours. Moreover, it suggests that P-cadherin aberrant expression may represent a relevant prognostic factor, being associated with an aggressive biological behaviour in feline mammary carcinomas, better than the reduction of E-cadherin expression. The prognostic value of P-cadherin expression in E-cadherin positive carcinomas in feline species should be evaluated in further investigation with follow-up studies.

## Abbreviations

H&E: Haematoxylin and eosin
WHO: World health organization
IHC: Immunohistochemistry
TBS: Tris-buffered saline
DAB: 3,3’-diaminobenzidine
BSA: Bovine serum albumin
P^+^: P-cadherin positive
P^−^: P-cadherin negative
E^+^: Preserved E-cadherin
E^−^: Reduced E-cadherin
PBS: Phosphate buffered saline
DIF: Double-label immunofluorescence
